# Modularity as a source of new morphological variation in the mandible of hybrid mice

**DOI:** 10.1186/1471-2148-12-141

**Published:** 2012-08-08

**Authors:** Sabrina Renaud, Paul Alibert, Jean-Christophe Auffray

**Affiliations:** 1 Laboratoire de Biométrie et Biologie Evolutive, UMR 5558, CNRS, Université Lyon 1, 69622, Villeurbanne, France; 2 Biogéosciences, CNRS, Université de Bourgogne, 21000, Dijon, France; 3 Institut des Sciences de l’Evolution, CNRS, Université Montpellier 2, 34095, Montpellier, France

**Keywords:** *Mus musculus*, House mouse, Mandible shape, Hybridization, Geometric morphometrics, Transgressive phenotypes

## Abstract

**Background:**

Hybridization is often seen as a process dampening phenotypic differences accumulated between diverging evolutionary units. For a complex trait comprising several relatively independent modules, hybridization may however simply generate new phenotypes, by combining into a new mosaic modules inherited from each parental groups and parts intermediate with respect to the parental groups. We tested this hypothesis by studying mandible size and shape in a set of first and second generation hybrids resulting from inbred wild-derived laboratory strains documenting two subspecies of house mice, *Musmusculus domesticus* and *Musmusculus musculus*. Phenotypic variation of the mandible was divided into nested partitions of developmental, evolutionary and functional modules.

**Results:**

The size and shape of the modules were differently influenced by hybridization. Some modules seemed to be the result of typical additive effects with hybrids intermediate between parents, some displayed a pattern expected in the case of monogenic dominance, whereas in other modules, hybrids were transgressive. The result is interpreted as the production of novel mandible morphologies. Beyond this modularity, modules in functional interaction tended to display significant covariations.

**Conclusions:**

Modularity emerges as a source of novel morphological variation by its simple potential to combine different parts of the parental phenotypes into a novel offspring mosaic of modules. This effect is partly counterbalanced by bone remodeling insuring an integration of the mosaic mandible into a functional ensemble, adding a non-genetic component to the production of transgressive phenotypes in hybrids.

## Background

How new morphological variation arises along the course of evolution is a key topic to understand how diversity is generated and maintained [1-3]. Morphological divergence can take place slowly with the accumulation of neutral genetic differences [4-7] leading to a certain amount of morphological differentiation roughly proportional to the genetic divergence. This slow pace of morphological evolution may be accelerated if selection occurs. Ecological shifts have been shown to make morphological evolution non-proportional to neutral genetic evolution [6,8,9].

In this context, hybridization between diverging populations has long been viewed as dampening differences, with hybrids expected to be in an intermediate position between the parental groups [10-13]. In many cases, however, hybrids display characteristic features that are not merely intermediate between parental traits but show new – termed transgressive –morphologies [14-17]. By increasing the range of morphological and ecological variation screened by selection [18,19], hybridization can thus contribute to the success of hybrids in new habitats [18] and participate to speciation [20,21]. In this context, studying how such transgressive phenotypes arise is of prime interest.

Progress in linking genetics and the development of morphological features in an “evo-devo” perspective might shed some further light on how morphological variation may arise. Most traits appear to follow a complex multigenic determinism [22-24]. In this context, additive genetic variation should favor in hybrids a “middle shape” half-way between both parents. In contrast, the combination of partial or total dominance, and overdominance effects at many loci, typical for multigenic characters, should contribute to the emergence of transgressive patterns in hybrids [22,23,25,26].

Some morphological features also appear to include different parts or “modules” characterized by a certain genetic independence from the others [25]. Because of this partly independent genetic determinism, the different modules are expected to diverge in a different way during a differentiation process. Such modular divergence has been shown, for instance, in the response to chromosomal rearrangements and isolation by distance [27]. Even in the case of very simple model of genetic determinism shaping each module, hybridization could produce an extended morphological variation outside the parental range by the mere combination of modules with different signatures. For instance, the combination of additive effects producing a shape half-way of parental groups on a first module, and effects analogue to monogenic dominance (i.e., producing a hybrid close to one of the parental group, an effect later on referred as ‘dominance’) with hybrids close to one parent for a second module and close to the other parent for a third module, would result in a fully new overall shape. This combinatory model could produce new shapes in hybrids departing from a mere intermediate shape between the parental groups in a simple but largely undocumented way.

**Figure 1:**
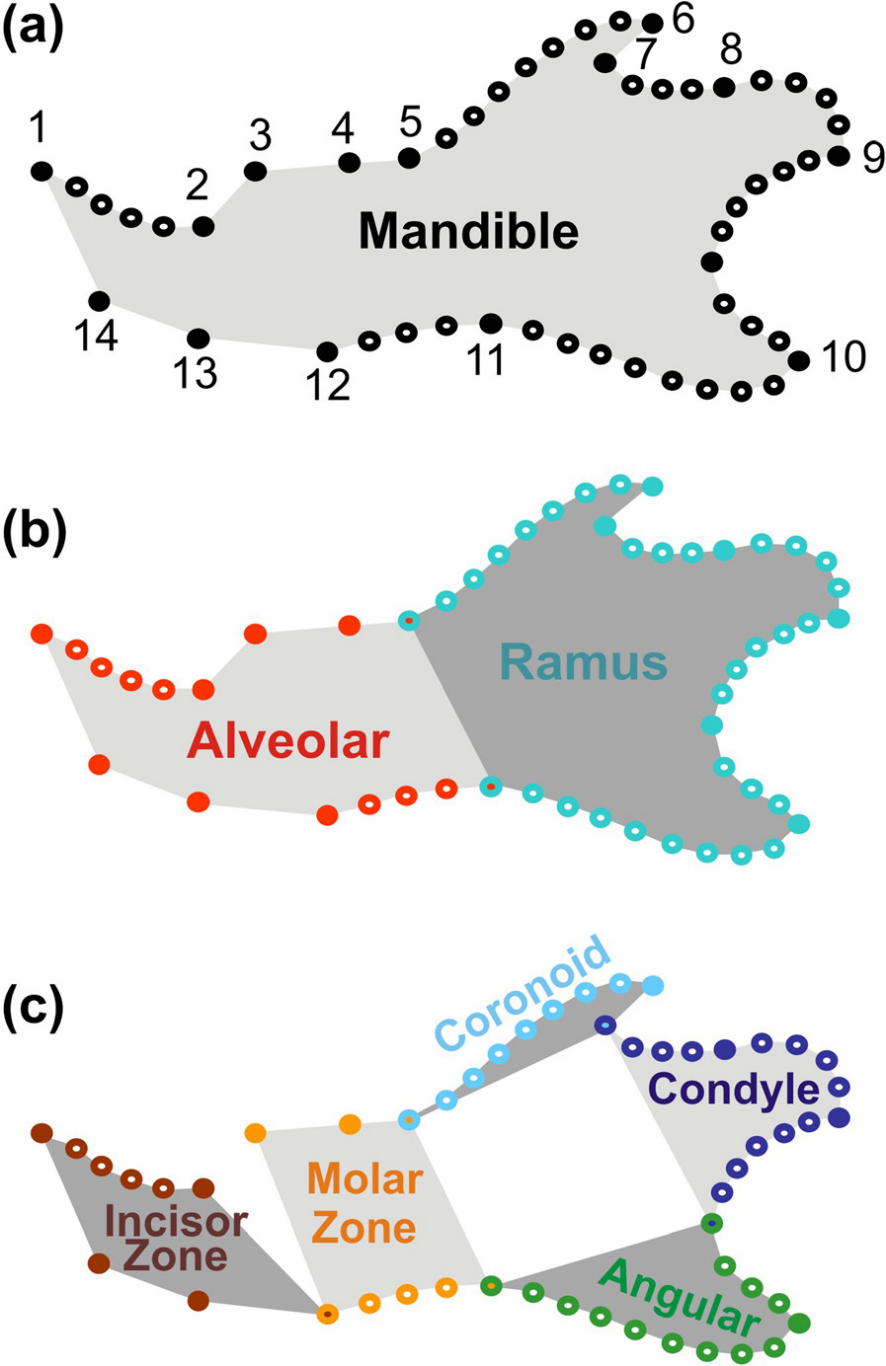
**Sampling of the mandible shape and definition of its modules.** (**a**) Whole mandible, sampled by 14 landmarks (dark dots) and eight curves, composed of sliding landmarks (open dots). (**b**) Partition of the mandible into two main modules (following Atchley et al. 1991): anteriorly, the alveolar part (in red) and posteriorly, the ramus (in blue). (**c**) Fine partition into five modules (following Monteiro et al. 2005): incisor-bearing zone (dark red), molar-bearing zone (orange) in the alveolar region; coronoid process (light blue), condyle (dark blue) and angular process (green) for the ramus region.

We investigated this hypothesis using a morphological feature that is emerging as an exemplary model for dissecting the genetic and developmental basis of evolution of complex morphological features: the mouse mandible [28,29] (Figure 1a). This structure presents several key advantages: the embryology of the mouse mandible is well known and has led to the identification of several modules and subsequent measures of the degree of integration between these parts confirmed their relative independence [30]. Developmental and genetic evidences [28,30] support the relative independence of the anterior, tooth-bearing zone or alveolar region, and of the posterior zone, or ramus, where most of the muscles are inserted (Figure 1b). Based on the functional and evolutionary evidence [31] a further partition, embedded in the first one, has been proposed composed of five modules: the anterior, incisor-bearing zone; the molar-bearing zone; and the three processes of the ramus, namely, in a top-down order the coronoid, condylar, and angular processes (Figure 1c). This complexity is still such that it can be successfully dissected, as opposed to more intricate features such as the skull [29].

The mouse also provides a well-known biological model for the study of hybridization. The hybrid zone across Europe between the two diverging subspecies *Mus musculus domesticus* and *Mus musculus musculus* is an exemplary case that has extensively been studied [32-36]. Laboratory strains of the two subspecies allow the documentation of hybridization effects on animals of known pedigree and in controlled conditions that discard possible environmental influences. The mandible of hybrid mice thus represents an optimal model to investigate the effect of modularity on the phenotypic output of hybridization.

In the context of hybridization, expectations regarding the pattern of divergence of the hybrids compared to the parents are the following. (1) Transgressive morphologies are likely to be frequent, if not even the rule, due to non-additive multigenic interactions [26]. The documentation of transgressive mandible shapes in F1 hybrids [37] already suggested that such processes occur in the hybridization of the two house mouse subspecies. (2) Transgressive morphologies in hybrids should be enhanced in the second generation and following ones due to complex genomic and epistatic interactions between the two diverging genomes, including the complementing effects of alleles accumulated in each parental group [38-41]. In the context of a modular response to hybridization, expectations would be further (3) to document a contrasted pattern of differentiation between hybrids and parents depending to the module; this should lead (4) to even more pronounced transgressive patterns in structures composed of several modules in comparison to those composed of a single module.

We thus investigated the mandible morphology in parental strains and their F1 and F2 hybrids, using geometric morphometric tools to consider how the overall variation of the mandible was the result of the variation of the modules composing it. The main predictions were the following. (1) Different modules should be characterized by a different response to hybridization reflecting a different genetic determinism. (2) Modularity could affect the shape of each module but also its size, which is known to increase in house mouse subspecific hybrids due to heterotic effects [42,43]. (3) The number of possible combinations of modules of different sizes and shapes increases from the second generation onwards, because of the recombination between the genomes and the increased number of allelic combinations. The transgression observed in F1 hybrids should thus be amplified in F2 hybrids whose phenotypic variance should be larger than in F1 hybrids.

## Methods

### Parental and hybrid mice

The mice from the two parental groups belong to strains bred from wild-trapped animals and conserved at the Wild Mus Genetic Conservatory at the Institut des Sciences de l’Evolution de Montpellier (ISEM, France). The western European house mouse *M. musculus domesticus* was represented by 33 mice from the strain WLA, derived from mice caught near Toulouse (France) in 1976. The Eastern European subspecies *M. musculus musculus* was represented by 24 mice from the strain PWK, derived from mice trapped in Prague (Czech Republic) in 1982.

38 F1 hybrids were bred from these parental groups, from both the WLA × PWK and the PWK × WLA pairs. In order to buffer among-pair differences and to increase sample size, all F1 were considered together. A total of 82 F2 hybrids were further bred from these F1 hybrids.

All 177 animals were bred in the same controlled conditions at the ISEM (Montpellier, France; authorization certificate 04143 (03/26/1991) to JCA of the Ministère de l’Agriculture et de la Forêt [French Ministry of Agriculture and Forest]). Both the PWK and WLA strains have been established and maintained by brother/sister matings at the Institut Pasteur (Paris, France) to obtain inbred wild-derived mouse strains and had been maintained in these breeding conditions for more than 50 generations at the time of the experiment.

### Data acquisition

The mandible was described by a set of 15 landmarks on the labial side of the mandible (Figure 1a), according to locations that have been shown to efficiently describe mandible shape variation [25,44].

1. Antero-dorsal border of the incisor alveolus;

2. Extreme of the diastema invagination;

3. Anterior edge of the molar tooth-row;

4. Boundary between the first and second molars along the edge of the mandibular bone;

5. Posterior intersection of the molar tooth-row with the coronoid surface;

6. Tip of the coronoid process;

7. Maximum of curvature between the coronoid and condylar processes;

8. Anterior edge of the articular surface of the condyle;

9. Posterior-most edge of the articular surface of the condyle;

10. Maximum of curvature on the curve between the condylar and angular process;

11. Tip of the angular process;

12. Dorsal-most point on the ventral border of the horizontal ramus;

13. Ventral-most point of inflexion of the ventral border of the horizontal ramus;

14. Posterior extremity of the mandibular symphysis;

15. Antero-ventral border of the incisive alveolus.

In order to get more detailed shape information on the curved zones of the mandibular bone, sliding landmarks were further registered (Figure 1a):

Curve 1: four sliding landmarks between landmarks 1 and 2 (antero-dorsal edge of the diastema invagination);

Curve 2: seven sliding landmarks between landmarks 5 and 6 (dorsal edge of the coronoid process);

Curve 3: three sliding landmarks between landmarks 7 and 8 (dorsal border of the condylar process);

Curve 4: four sliding landmarks between landmarks 8 and 9 (edge of the articular surface of the condyle);

Curve 5: five sliding landmarks between landmarks 9 and 10 (posterior border of the condylar process);

Curve 6: three sliding landmarks between landmarks 10 and 11 (postero-dorsal edge of the angular process);

Curve 7: eight sliding landmarks between landmarks 11 and 12 (ventral border of the angular process);

Curve 8: three sliding landmarks between landmarks 12 and 13 (ventral border of the molar zone).

This data set (15 landmarks and 37 sliding landmarks) describes the complete mandible. It has been digitized on the left mandibles (or exceptionally on a mirror image of the right mandible when the left one was damaged) put flat on their lingual side using TPSdig2 [45]. The number of points documenting each curve was chosen to provide an approximately similar coverage of each zone of the mandible. The antero-ventral zone of the mandible was not covered by curves because it is relatively straight and influenced by how the two hemi-mandibles were separated at the symphysis.

### Shape analyses

This data set was split into several subsets describing the modules of the mandible. As mentioned above, two main modules were recognized in the mouse mandible [30]: the alveolar region and the ascending ramus (Figure 1b). These two regions correspond to landmarks 1, 2, 3, 4, 5, 12, 13, 14 and 15 together with curves 1 and 8 for the alveolar region and landmarks 5, 6, 7, 8, 9, 10, 11 and 12 and with curves 2, 3, 4, 5, 6 and 7 for the ramus.

A secondary partition into smaller modules was also proposed [31,44]: anterior alveolar ('incisor zone'), posterior alveolar (insertion zone of the molars), as well as the coronoid, condylar and angular processes. We therefore split the data-set into 5 subsets (Figure 1c):

1. Incisor zone (anterior alveolar): landmarks 1, 2, 14 and 15 together with curve 1;

2. Molar zone (posterior alveolar): landmarks 3, 4, 5, 12 and 13 together with curve 8;

3. Coronoid: landmarks 5, 6 and 7 together with curve 2;

4. Condyle: landmarks 7, 8, 9 and 10 together with curves 3, 4 and 5;

5. Angular: landmarks 10, 11 and 12 together with curves 6 and 7.

For each data-set, a Procrustes superimposition taking into account the specificity of the sliding landmarks was performed using TPSrelw [46]. Using this method, configurations of landmarks and sliding landmarks were scaled, translated and rotated in order to minimize the summed squared distance between corresponding landmarks [47]. The size measure used for scaling the configuration is the square root of the summed squared distances between landmarks and their center of gravity. This measure of size, so-called “centroid size” [48], was used thereafter as size estimates of the different configurations.

The Cartesian coordinates obtained after this superimposition constitute the shape coordinates that can be analyzed using multivariate statistics. The total variance can be summarized using a standard principal component analysis (PCA).

Shape differences among groups were tested for each partition using multivariate analyses of variance (MANOVA) performed on the axes of the PCA explaining more than 5% of the total variance.

In order to have an average indicator of the morphological distance between the parental strains, and understand how F1 and F2 hybrids were positioned within this framework, consensus shapes were computed for each of the four groups (*domesticus* parental strain WLA, *musculus* parental strain PWK, F1 and F2 hybrids) separately. Distances between these consensus shapes were computed using TPSsmall [49]. In the absence of transgression, hybrids should be in a strictly intermediate position between the parental strains, and hence the sum of the distances between the hybrids and each one of the two parental strains should be equal to the distance between the two parental strains: i.e. d(F1, WLA) + d(F1, PWK) = d(WLA, PWK).

The degree of transgression was then assessed as the degree of deviation of the hybrids from this theoretic expectation, hence for F1 hybrids: d(F1, WLA) + d(F1, PWK) – d(WLA, PWK) expressed as a percentage of the inter-parental strains distance d(WLA, PWK).

The degree of closeness to a parental strain, pointing to dominance-like pattern, was estimated by assessing the difference in the distance to one parental strain with respect to the average distance between the hybrids and the two parental strains, hence for F1 hybrids: [d(F1, WLA) + d(F1, PWK)]/2 – d(F1, WLA), expressed as the percentage of the average distance of hybrids to the parental strains [d(F1, WLA) + d(F1, PWK)]/2. Positive values then indicate closeness to WLA, and negative values closeness to PWK.

### Size analysis

The centroid size (square root of the summed squared distances between the landmarks and their center of gravity) was used as size estimates of the different configurations. Among-group differences were tested by one-way analyses of variance (ANOVA) completed by two-by-two post hoc Tukey tests. Possible relationships with shape were investigated using regressions of size vs. the first two multivariate axes, cumulating most of the variance, in order to evaluate if the shape pattern could be attributed to an allometric effect. This analysis was performed for the whole mandible and each module separately, considering then the size of this particular module as the size estimate.

### Patterns of integration

Two complementary approaches were used to determine if the variation in one module was related to the variation in another one [50]. Shape distances between specimens were computed using TPSsmall [49]. The distance matrices were then compared using a Mantel test. A significant relationship between the inter-individual variation in two modules indicates their integration, whereas a non-significant relationship points to an independence of the two modules.

An alternative approach was to calculate the strength of the association between modules using RV coefficients [51]. This coefficient corresponds to the sum of the squared covariances between two sets of variables, divided by the total amount of variation in the two sets of variables [52]. Here, each set of variables corresponded to a module of the mandible (the PC scores being obtained after a Procrustes superimposition performed for each module separately). The significance of the association was tested by comparing the observed RV coefficient to a distribution obtained by permuting each row (individual) separately in each of the two sets of variables (999 permutations). These tests were performed using the ade4 package [53].

Finally, the shape variation corresponding to the association between modules was visualized using Partial Least Squares (PLS) analyses [54]. This multivariate technique splits the covariance between two sets of variables, here the different modules of the mandible, into principal axes, one for each set of variables. The strength of the covariation between the two sets can be evaluated by the amount of variance explained by a given PLS axis, and by way scores on the PLS first axis of the first set are correlated with the scores on the PLS axis of the second set.

## Results

### Size differences

**Figure 2:**
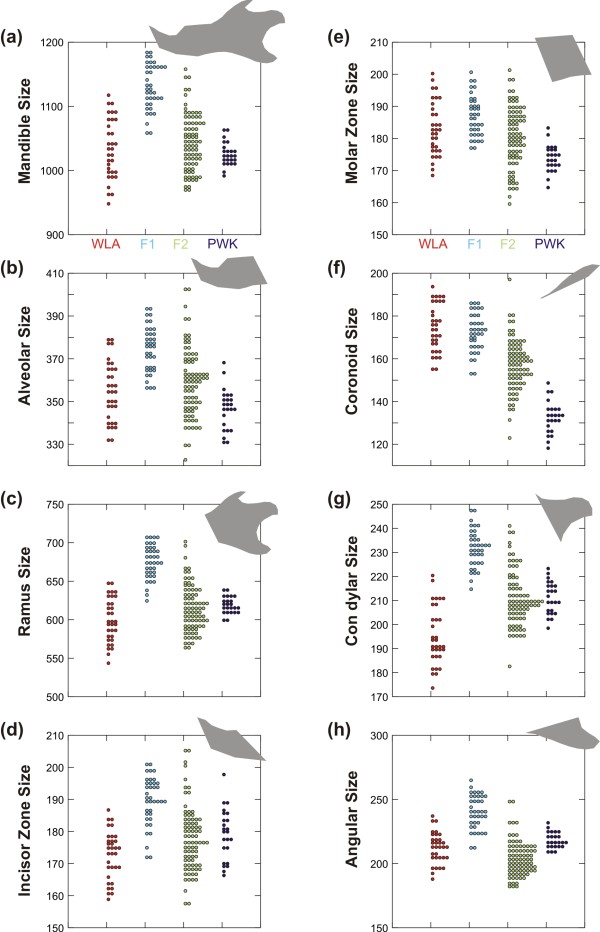
**Size variations between parental strains and their hybrids.** (**a**) Whole mandible. (**b**, **c**) The two main modules. (**d**, **e**, **f**, **g**, **h**) The five local modules. The *domesticus* parental strain WLA is represented in red, the *musculus* parental strain PWK in dark blue, the F1 hybrids in light blue and the F2 hybrids in green. Each dot corresponds to a specimen. In grey, the overall shape of the involved region.

Differences in size among groups were highly significant for the whole mandible and each module considered separately (Figure 2; Table 1). Parental strains (WLA and PWK) were not significantly different in size for the whole mandible, the alveolar region, and the angular process. Significant differences existed for other parts, but in different directions: the WLA were larger than the PWK for the molar zone and the coronoid process (P < 0.001). The opposite was observed for the ramus (P = 0.024), the incisor region (P = 0.036) and the condylar process (P < 0.001).

**Table 1:** Size differences between parental strains and their hybrids for each module of the mandible

	**4 groups**	**WLA-PWK**	**F1-WLA**	**F1-PWK**	**F2-WLA**	**F2-PWK**	**F1-F2**
Mandible	**0.000**	0.862	**0.000**	**0.000**	0.628	0.200	**0.000**
Alveolar	**0.000**	0.119	**0.000**	**0.000**	0.391	**0.000**	**0.000**
Ramus	**0.000**	*0.024*	**0.000**	**0.000**	*0.016*	0.922	**0.000**
Incisor Zone	**0.000**	*0.036*	**0.000**	**0.000**	0.031	0.908	**0.000**
Molar Zone	**0.000**	**0.000**	0.164	**0.000**	0.372	*0.002*	**0.000**
Coronoid	**0.000**	**0.000**	0.854	**0.000**	**0.000**	**0.000**	**0.000**
Condylar	**0.000**	**0.000**	**0.000**	**0.000**	**0.000**	1.000	**0.000**
Angular	**0.000**	0.187	**0.000**	**0.000**	*0.006*	**0.000**	**0.000**

First column (“4 groups”): P values of an ANOVA on the size (estimated by the centroid size of the given module of the mandible) of the two parental groups, and the F1 and F2 hybrids. Next column: P values of the two-by-two post hoc Tukey tests. In italics significant probabilities (P < 0.05), in bold highly significant probabilities (P < 0.001).

F1 hybrids were significantly larger than their parents in all cases (P < 0.001) except for the molar region and the coronoid where F1 hybrids displayed a larger size than the PWK parents, but the same size as the WLA parents. They differed in all cases from the F2 hybrids (P < 0.001), which had a wider distribution in size and were overall smaller than the F1 for all mandible parts. F2 hybrids frequently differed from the parental strains, except for the whole mandible. They differed from both strains for the coronoid process (P < 0.001), being of intermediate size, and for the angular process for which they displayed the smallest size (vs. WLA P = 0.006, vs. PWK P < 0.001). They were similar in size to the WLA parents for the alveolar region and the molar zone, and larger than the PWK parents (alveolar: P < 0.001; molar: P = 0.002). They were in turn similar in size to PKW parents for the ramus, the incisor zone, and the condylar process. In these cases, the F2 hybrids were larger than the WLA parents (ramus: P = 0.016; incisor: P = 0.031; condylar: P < 0.001).

### Shape variation

**Figure 3:**
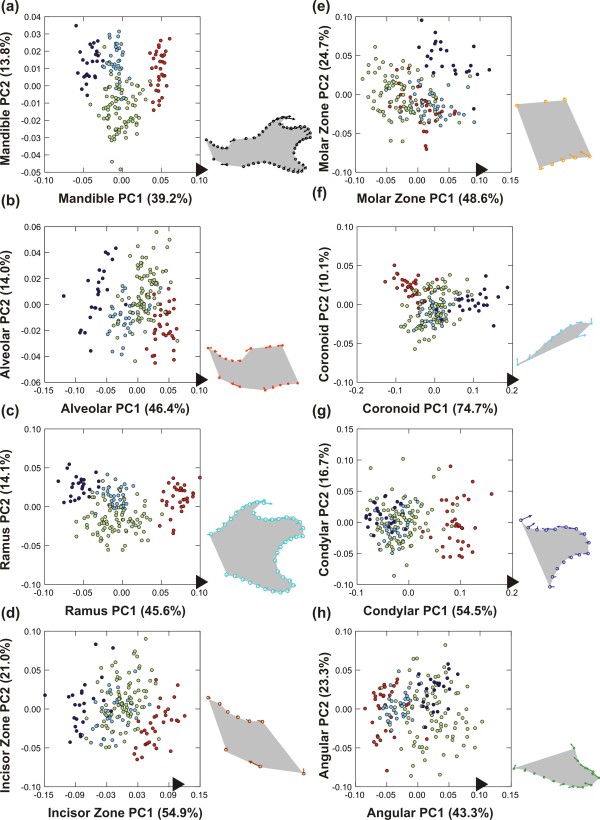
**Shape variation between parental strains and their hybrids.** (**a**) Whole mandible. (**b**, **c**) The two main modules. (**d**, **e**, **f**, **g**, **h**) The five local modules. The total morphological variation is represented in the morphological space defined by the first two axes of a principal component analysis on the superimposed coordinates of landmarks and sliding landmarks of the involved region. The percentage of variance explained by each axis is provided in brackets. The *domesticus* parental strain WLA is represented in red, the *musculus* parental strain PWK in dark blue, the F1 hybrids in light blue and the F2 hybrids in green. Each dot corresponds to one specimen. Next to each graph, the visualization of the shape change occurring along the first axis, with vectors representing the difference between the consensus shape (origin of the morphological space) and the score along the axis marked by a dark arrow.

Shape differences among groups were highly significant in all cases (P < 0.0001). Patterns of differentiation between parental groups and hybrids were visualized on the first two axes of the PCA performed on the shape variables (Figure 3). Despite the high number of variables, in each case, the expected pattern of differentiation is expected to be summarized onto three main axes: one displaying difference between parental groups, a second characterizing the transgressive direction of hybrids, and a third expressing intra-group variation. Percentages of variance of the five first axes are provided (Table 2) to assess the importance of the signal expressed on each axis.

**Table 2:** Percentage of variance explained by successive shape axes for each module of the mandible

	**PC1**	**PC2**	**PC3**	**PC4**
Mandibule	39.2	13.8	9.3	7.6
Alveolar	46.4	14.0	12.0	7.8
Ramus	45.6	14.1	10.0	9.5
IncisorZ	54.9	21.0	9.4	6.2
MolarZ	48.6	24.7	10.6	7.0
Coronoid	74.7	10.1	6.5	4.9
Condyle	54.5	16.7	10.8	4.5
Angular	43.3	23.3	16.5	6.8

The shape of each module was analyzed using a principal component analysis on the Cartesian coordinates of the landmarks and sliding landmarks after a Procrustes superimposition. Difference between parental groups is expected to be displayed on the first axis and transgression characterizing hybrids on the second axis. The percentage of variance explained by the successive axes allows evaluating the relative importance of the signal displayed on each axis.

In most of the cases, the first axis (representing from 40 to 55% of the total variance) corresponded, as expected, to the difference between both parental strains with the F1 and F2 hybrids in between (Figure 3). The second axis expressed the divergence characteristic of the hybrids, and hence pointed out the transgression effect.

F1 hybrids overall appeared to be slightly transgressive, but much less than F2 hybrids were. This was in apparent contrast with former results reported on the whole mandible that had evidenced a pronounced transgressive pattern in F1 hybrids [37]. This discrepancy might be attributed to different methods for investigating shape variation (outline analysis in the former study vs. landmarks and sliding landmarks in the present one). This interpretation was undermined by a landmark + sliding landmark analysis including F1 hybrids only that delivered results very similar to the outline analysis, with a transgressive signature of the hybrids (data not shown). More likely, the strong transgression of F2 hybrids was overwhelmingly driving the pattern of differentiation and made the signature of F1 hybrids more difficult to identify. This pattern characterized the analysis of the whole mandible and of most modules, but some modules deviated from this general pattern.

Regarding the whole mandible (Figure 3a), the differentiation followed the exemplary pattern of WLA and PWK parental strains opposed along the first axis, hybrids having an intermediate position along this first axis and a slightly divergent one (especially F2) along the second axis. A migration towards the WLA *domesticus* pole along the first axis involves changes distributed overall on the mandible: a posterior elongated, more curved coronoid process, a reduced condyle, a slight ventro-dorsal contraction of the angular process, and a molar zone shifted anteriorly.

When considering the anterior part of the mandible only (alveolar region) (Figure 3b), the pattern was overall similar, with a first axis opposing both parental strains, hybrids being intermediate. The WLA individuals were characterized by an anterior shift of the zone of the molar insertion, and a strong anterior shift of the ventral-most point of inflexion of the ventral border of the horizontal ramus (landmark 13).

The pattern was similar when considering the posterior part of the mandible (ramus) (Figure 3c). Yet, the F1 hybrids did not occupy a strictly intermediate position between the parental strains but tended to be closer to the PWK parents, characterized by an antero-posterior reduction of the coronoid and the angular processes.

When considering the mandible as a group of five modules, some patterns tended to diverge from this exemplary model where hybrids occupied an intermediate position along the first shape axis between the parental strains and were more or less transgressive along the second shape axis. This was still true for the anterior-most part (incisor zone, Figure 3d) but the pattern was drastically different for the molar zone (Figure 3e). Hybrids tended to be clustered with the WLA parents. This was also the case for the size of this module. The WLA and hybrids were characterized by an anterior pinching of the zone.

Regarding the posterior part of the mandible, the coronoid process (Figure 3f) exhibited the usual pattern of differentiation (hybrids intermediate, slightly transgressive when compared to the parental strains). This situation contrasted with its pattern of size differentiation where it displayed a size similar to the one of the WLA parents. The shape differentiation in this zone corresponded to a change in the curvature of the process, it being more convex for the WLA.

In contrast, the condyle displayed a pattern where hybrids were close to one of the parental strains (‘dominance effect’), but unlike the molar zone, in favor of the PWK (Figure 3g). WLA tended to have an antero-posteriorly slightly reduced condyle that was also dorsally expanded.

Finally, the angular process (Figure 3h) displayed an intriguing pattern. F1 hybrids were in an intermediate position, but slightly closer to the WLA parents whereas the highly variable F2 hybrids seemed to overlap more with the PWK parents. Changes in this zone corresponded to a posterior contraction of the angular process (PWK and F2) whereas the WLA exhibited a rather dorsally constricted process.

### Integration and dominance in the different modules

**Figure 4:**
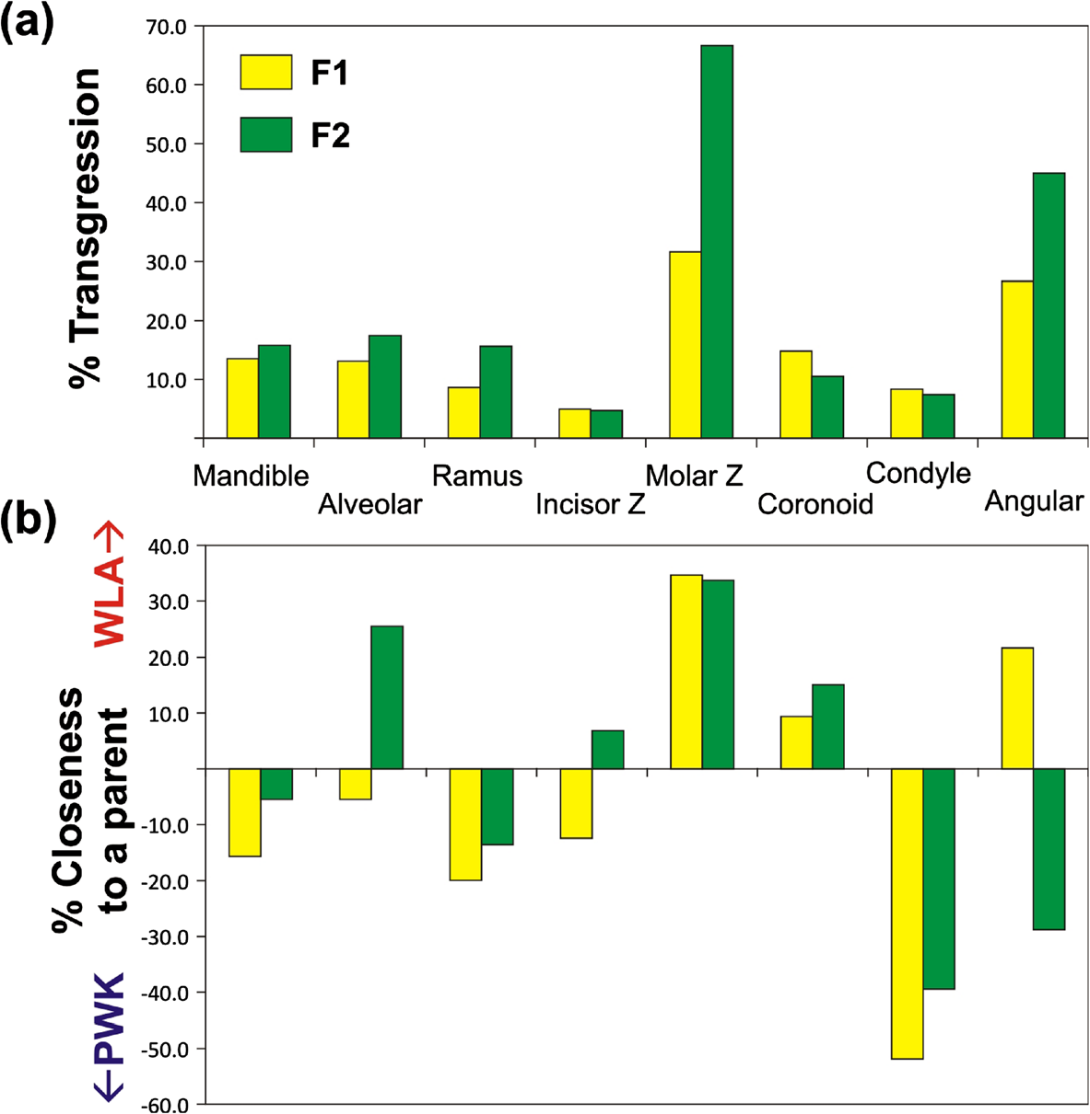
**Transgression in hybrid shape and closeness to parental strains for each partition of the mandible.** (**a**) Degree of transgression of F1 and F2 hybrids. In the absence of transgression, hybrids should be in a strictly intermediate position between the parental strains, and hence the sum of the distances between the hybrids and each one of the two parental strains should be equal to the distance between the two parental strains: e.g. d(F1, WLA) + d(F1, PWK) = d(WLA, PWK). The degree of transgression is thus given by the extent to which the hybrids differed from this theoretic expectation, hence for F1 hybrids: d(F1, WLA) + d(F1, PWK) – d(WLA, PWK) expressed as a percentage of the inter-parental strains distance d(WLA, PWK). (**b**) Degree of closeness to the parental strains. The degree of closeness to a parental strain was estimated by assessing the extent to which the distance to one parental strain was smaller than the average distance between the hybrids and the two parental strains, hence for F1 hybrids: [d(F1, WLA) + d(F1, PWK)]/2 – d(F1, WLA), expressed as the percentage of the average distance of hybrids to the parental strains [d(F1, WLA) + d(F1, PWK)]/2. Positive values thus indicate closeness to the WLA, and negative values to the PWK parental group. Consensus (average) shape was calculated for each one of the four groups (*domesticus* WLA, *musculus* PWK, F1 and F2 hybrids) and morphological distances were computed based on a Procrustes superimposition of these four items.

The way the different modules responded to hybridization was summarized by comparing average shapes (consensus) of the F1 and F2 hybrids to those of the parental strains. Morphological distances between the four groups (Procrustes distances, calculated after a Procrustes superimposition of the four consensus shapes) were computed for each partition of the mandible. The expectation of hybrids being in a strictly intermediate position between both parental groups would correspond to a distance between the hybrids and each one of the parents equal to half the distance between the parents. Distances larger than this value indicate transgression (if both hybrid-parents are larger than expected). Hybrids closest to one parental strain would correspond to one hybrid-parent distance much smaller than the other. The relative degree of transgressivity of F1 and F2 hybrids (Figure 4a) and the degree of closeness to the parental shapes (Figure 4b) were thus estimated based on the distance among groups.

Transgressivity was estimated as the amount in which the cumulated distance between hybrids and both parents exceeded the theoretic distance without transgression (inter-parents distance). Shapes composed of several modules, i.e. the whole mandible, the alveolar region and the ramus, tended to display slightly higher levels of transgressivity (> 15% in F2 hybrids) than single modules such as the incisor zone (< 5%) and the condyle (< 10%) (Figure 4a). Exceptions to this were the molar zone and the angular process, both displaying very strong transgressive patterns.

The closeness to one parental strain, estimating the degree of ‘dominance’, was assessed as the contribution of this hybrid-parent distance to the cumulated distance between hybrids and both parents (Figure 4b). The most striking signatures correspond to the molar zone, with a pronounced dominance of the WLA parental shape, and the condylar process and to a lesser degree the ramus, with a dominance of the PWK parental shape.

### Size and shape relationships

The first axis of the multivariate analysis usually represents the differentiation of the parental strains. Considering the whole mandible, this axis was not related to size (R^2^ = 0.008, P = 0.232). Considering the modules separately, a significant relationship (P ≤ 0.001) with size was evidenced for the ramus (R^2^ = 0.063), the incisor part (R^2^ = 0.090), the coronoid (R^2^ = 0.461), the condyle (R^2^ = 0.225) and the angular process (R^2^ = 0.190). Except for the coronoid process, the variance explained is low and the observed correlation might simply be the result of a strong differentiation in size and of the four groups accidentally leading to a significant relationship.

The second multivariate axis in most cases represents the transgressive signal. A significant correlation with size (P ≤ 0.001) was evidenced for the whole mandible (R^2^ = 0.104), the alveolar part (R^2^ = 0.061) and the incisor part (R^2^ = 0.065). Here again the percentage of variance explained is low and the few significant relationships might be due to the congruence of a strong heterosis and transgressive effects in hybrids. Overall, these results do not support the idea that the transgressive effect, when documented, could be a by-product driven by allometric coupling of a heterotic size increase, confirming the results of a previous study on the whole mandible [37].

### Integration among modules

It is clear from the previous results that the mandible indeed responded to hybridization in a fully modular way. Despite this modularity, its functional efficiency needs to be maintained. We therefore investigated the patterns of integration between modules, and hence the way they covary with each other. This was estimated within each group, by comparing the matrices of distance based on each partition of the mandible (Table 3) and by RV coefficients (Table 4). Overall, both methods provided rather comparable results.

**Table 3:** Mantel correlations among partitions and modules of the mandible

**WLA**	**Mandib**	**Alveolar**	**Ramus**	**IncisorZ**	**MolarZ**	**Coronoid**	**Condyle**	**Angular**
Mandib	—	**1.00**	**1.00**	**0.99**	**1.00**	**1.00**	**1.00**	**1.00**
Alveolar	**0.390**	—	0.681	**1.00**	**1.00**	0.78	0.44	0.77
Ramus	**0.973**	0.041	—	0.54	0.89	**1.00**	**1.00**	**1.00**
IncisorZ	**0.173**	**0.685**	0.008	—	**0.99**	0.76	0.41	0.67
MolarZ	**0.314**	**0.663**	0.112	**0.198**	—	0.92	0.36	0.88
Coronoid	**0.307**	0.080	**0.378**	0.061	0.139	—	0.94	**0.96**
Condyle	**0.478**	-0.015	**0.559**	-0.020	-0.034	0.156	—	**0.99**
Angular	**0.518**	0.063	**0.612**	0.036	0.101	**0.152**	**0.211**	—
PWK	Mandib	Alveolar	Ramus	IncisorZ	MolarZ	Coronoid	Condyle	Angular
Mandib	—	**1.00**	**1.00**	**1.00**	**1.00**	**1.00**	0.78	**1.00**
Alveolar	**0.542**	—	0.87	**1.00**	**1.00**	0.60	0.07	**0.97**
Ramus	**0.809**	0.121	—	0.43	**1.00**	**1.00**	**0.98**	**1.00**
IncisorZ	**0.401**	**0.830**	-0.020	—	**0.979**	0.17	0.16	0.51
MolarZ	**0.470**	0.643	**0.277**	**0.228**	—	**0.99**	0.09	**1.00**
Coronoid	**0.360**	0.029	**0.411**	-0.116	**0.245**	—	0.38	0.88
Condyle	0.075	-0.143	**0.199**	-0.106	-0.121	-0.029	—	0.32
Angular	**0.384**	**0.199**	**0.436**	0.004	**0.383**	0.126	-0.043	—
F1	Mandib	Alveolar	Ramus	IncisorZ	MolarZ	Coronoid	Condyle	Angular
Mandib	—	**1.00**	**1.00**	**1.00**	**1.00**	**1.00**	**1.00**	**1.00**
Alveolar	**0.586**	—	**1.00**	**1.00**	**1.00**	0.57	0.90	**1.00**
Ramus	**0.904**	**0.393**	—	0.85	**1.00**	**1.00**	**1.00**	**1.00**
IncisorZ	**0.244**	**0.618**	0.076	—	0.88	0.50	0.89	0.43
MolarZ	**0.482**	**0.751**	**0.384**	0.095	—	0.28	0.45	**1.00**
Coronoid	**0.251**	0.016	**0.328**	0.000	-0.052	—	0.53	0.88
Condyle	**0.321**	0.111	**0.374**	0.099	-0.011	0.006	—	0.28
Angular	**0.413**	**0.340**	**0.476**	-0.014	**0.506**	0.100	-0.049	—
F2	Mandib	Alveolar	Ramus	IncisorZ	MolarZ	Coronoid	Condyle	Angular
Mandib	—	**1.00**	**1.00**	**1.00**	**1.00**	**1.00**	**1.00**	**1.00**
Alveolar	**0.377**	—	**1.00**	**1.00**	**1.00**	0.71	**0.95**	**0.99**
Ramus	**0.883**	**0.200**	—	**0.99**	**1.00**	**1.00**	**1.00**	**1.00**
IncisorZ	**0.239**	**0.681**	**0.119**	—	**0.99**	0.71	**0.99**	0.69
MolarZ	**0.321**	**0.667**	**0.203**	**0.141**	—	0.43	0.80	**1.00**
Coronoid	**0.244**	0.028	**0.281**	0.029	-0.010	—	0.07	0.48
Condyle	**0.328**	**0.097**	**0.412**	**0.144**	0.049	-0.078	—	0.92
Angular	**0.410**	**0.116**	**0.467**	0.027	**0.194**	-0.002	0.074	—

The inter-individual Procrustes distances were computed among the four groups (WLA and PWK parents, F1 and F2 hybrids) for the complete mandible and its partition into two (alveolar + ramus) and five (incisor zone, molar zone, coronoid, condylar and angular processes) modules. The degree of integration was evaluated by Mantel-t tests providing a coefficient of matrix correlation R (below the diagonal) and the probability that correlation between random matrices is less than observed (above the diagonal). In bold, significant correlations at 5% (P > 0.95%).

**Table 4:** Association among partitions and modules of the mandible, estimated by RV coefficients

**WLA**	**Mandib**	**Alveolar**	**Ramus**	**IncisorZ**	**MolarZ**	**Coronoid**	**Condyle**	**Angular**
Mandib	_	**0.001**	**0.001**	**0.001**	**0.001**	**0.002**	**0.001**	**0.001**
Alveolar	**0.558**	_	**0.006**	**0.001**	**0.001**	0.133	0.129	**0.038**
Ramus	**0.908**	**0.285**	_	0.078	**0.005**	**0.001**	**0.001**	**0.001**
IncisorZ	**0.330**	**0.752**	0.177	_	**0.007**	0.379	0.065	0.219
MolarZ	**0.441**	**0.687**	**0.273**	**0.255**	_	0.077	0.371	**0.020**
Coronoid	**0.360**	0.168	**0.389**	0.093	0.161	_	0.085	**0.003**
Condyle	**0.556**	0.179	**0.616**	0.170	0.112	0.160	_	**0.022**
Angular	**0.593**	**0.209**	**0.670**	0.120	**0.203**	**0.314**	**0.213**	_
**PWK**	Mandib	Alveolar	Ramus	IncisorZ	MolarZ	Coronoid	Condyle	Angular
Mandib	_	**0.001**	**0.001**	**0.001**	**0.001**	**0.002**	**0.002**	**0.004**
Alveolar	**0.625**	_	**0.038**	**0.001**	**0.001**	0.204	0.855	0.092
Ramus	**0.885**	**0.330**	_	0.845	**0.001**	**0.001**	**0.001**	**0.001**
IncisorZ	**0.456**	**0.834**	0.154	_	**0.004**	0.784	0.994	0.535
MolarZ	**0.539**	**0.752**	**0.368**	**0.365**	_	0.079	0.566	**0.018**
Coronoid	**0.429**	0.178	**0.425**	0.081	0.200	_	0.505	0.351
Condyle	**0.460**	0.159	**0.556**	0.086	0.147	0.165	_	0.458
Angular	**0.398**	0.227	**0.476**	0.118	**0.276**	0.136	0.179	_
**F1**	Mandib	Alveolar	Ramus	IncisorZ	MolarZ	Coronoid	Condyle	Angular
Mandib	_	**0.001**	**0.001**	**0.001**	**0.001**	**0.001**	**0.001**	**0.001**
Alveolar	**0.661**	_	**0.001**	**0.001**	**0.001**	**0.037**	**0.024**	**0.001**
Ramus	**0.921**	**0.495**	_	**0.027**	**0.001**	**0.001**	**0.001**	**0.001**
IncisorZ	**0.349**	**0.717**	**0.202**	_	0.145	0.060	**0.020**	0.349
MolarZ	**0.602**	**0.696**	**0.529**	0.111	_	0.174	0.251	**0.001**
Coronoid	**0.301**	**0.167**	**0.350**	0.124	0.091	_	0.105	0.213
Condyle	**0.402**	**0.198**	**0.473**	**0.183**	0.102	0.131	_	0.371
Angular	**0.491**	**0.367**	**0.527**	0.102	**0.509**	0.099	0.111	_
**F2**	Mandib	Alveolar	Ramus	IncisorZ	MolarZ	Coronoid	Condyle	Angular
Mandib	_	**0.001**	**0.001**	**0.001**	**0.001**	**0.001**	**0.001**	**0.001**
Alveolar	**0.398**	_	**0.001**	**0.001**	**0.001**	0.461	**0.012**	**0.025**
Ramus	**0.885**	**0.201**	_	0.123	**0.001**	**0.001**	**0.001**	**0.001**
IncisorZ	**0.218**	**0.716**	0.091	_	**0.001**	0.769	**0.019**	0.479
MolarZ	**0.327**	**0.678**	**0.172**	**0.184**	_	0.140	0.120	**0.002**
Coronoid	**0.197**	0.050	**0.229**	0.027	0.054	_	0.392	0.173
Condyle	**0.441**	**0.113**	**0.540**	**0.096**	0.071	0.042	_	**0.006**
Angular	**0.419**	**0.105**	**0.460**	0.050	**0.112**	0.055	**0.082**	_

Among each one of the four groups (WLA and PWK parents, F1 and F2 hybrids), the strength of association between two modules of a partition of the mandible was evaluated using RV coefficients. Observed RV values are provided below the diagonal. Probabilities of significance were obtained by comparing observed values to separate permutations of individuals on each of the modules (999 permutations, P above the diagonal). In bold, significant correlations at 5%.

**Figure 5:**
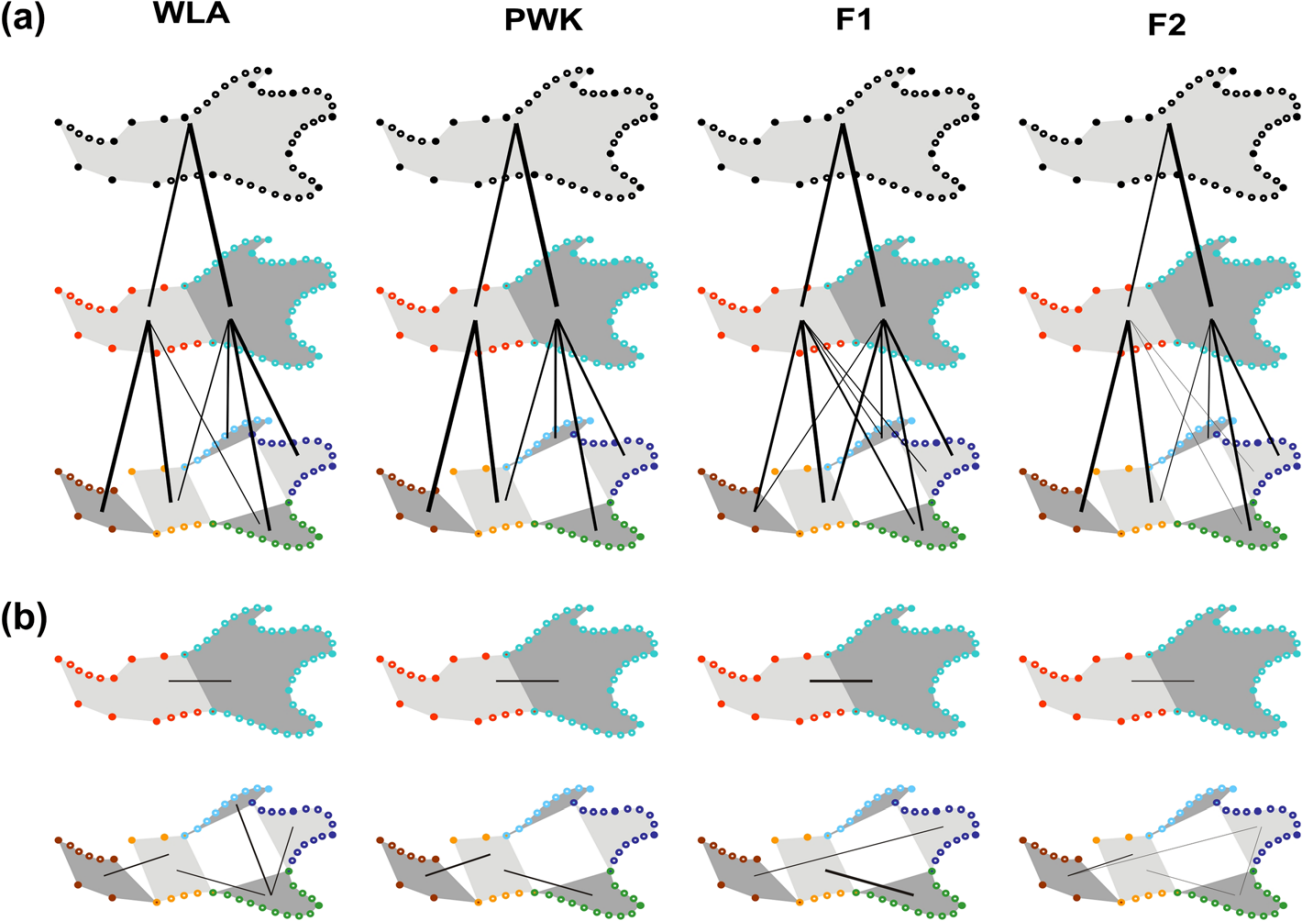
**Shape integration between modules of the mandible.** (**a**) Contribution of nested modules to the variation of the ensemble. The inter-individual distances were calculated for each partition of the mandible (above, total mandible; middle: two main modules; below: five local modules). The correlations between distances pointed to a strong contribution of a part (module) to the ensemble (main module or whole mandible) it contributes to. (**b**) Integration between modules. Inter-individual distances were calculated and compared between modules of a same partition of the mandible. Significant relationships pointed to integration between two modules. The lines represent significant correlations and their width is proportional to the strength of the relationship (RV coefficient, Table 4).

The first kind of comparisons involved modules that are subsets of others (Figure 5a). The variation of the whole mandible tended to be more related to the ramus than to the alveolar region. This was probably a mere consequence of sampling, since more landmarks and sliding landmarks sampled the former than the latter. This sampling reflected the mere geometry of the mouse mandible, displaying a relatively larger posterior part than an anterior one. Regarding the further partition of the mandible into five modules, the variation in the alveolar region was highly related to the variation in its two subsets (incisor and molar zones). The variation of the ramus was less related to the variation of each of its subsets (coronoid, condylar and angular processes) and displayed less congruence among groups. This suggests that the pattern of variation of the ramus was an emergent feature of the combination of the subsets, possibly because the modules are more loosely related with each other, mere appendices around a central zone that was not analyzed *per se* because of the lack of points defining it independently of landmarks shared with other modules.

**Figure 6:**
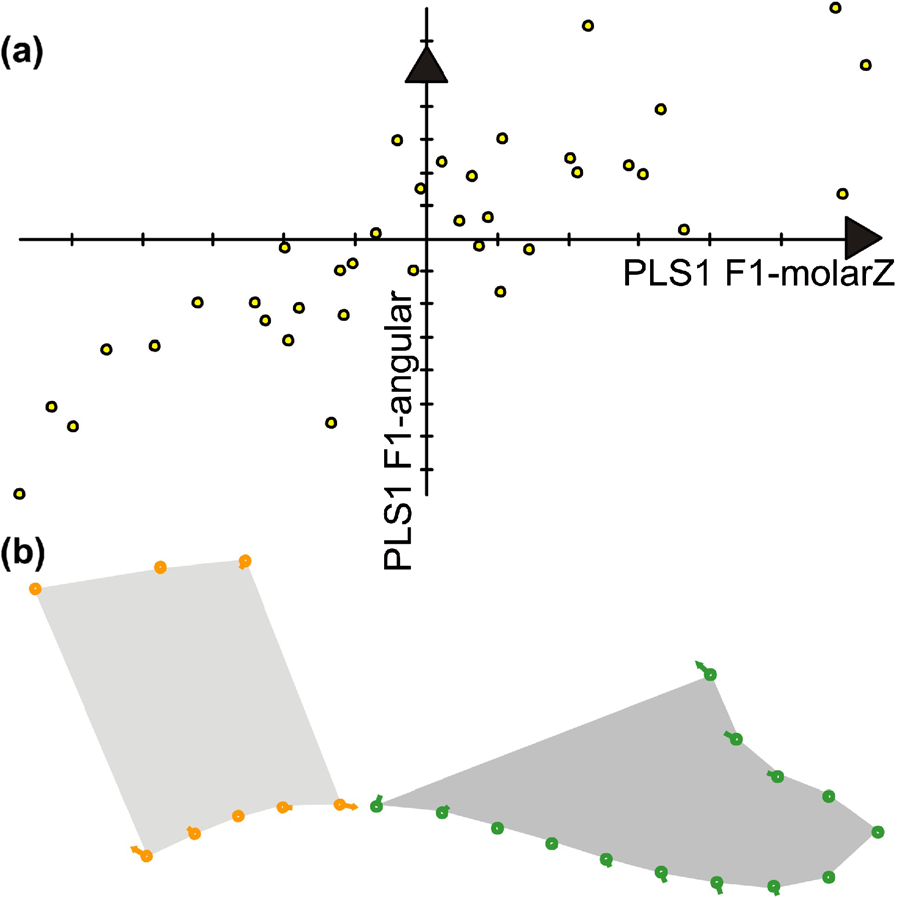
**Shape covariation between the molar zone and the angular process illustrated in F1 hybrids.** (**a**) The covariation between the molar zone and the angular process was exemplified in the group of F1 hybrids. A Partial Least Squares analysis (PLS) extracted a first dimension of covariance expressing 96.6% of the total covariance between the two modules. Scores on the first PLS axis corresponding to the molar zone and to the angular axis (x and y axes on the scatterplot) were highly correlated (R = 0.810), showing that a change in the molar zone was consistently associated with a change in the angular process. (**b**) Shape changes along the axes, depicted by vectors pointing from the consensus to a shape change corresponding to a covariation of 0.070 along PLS1 of the molar zone and of 0.062 along PLS1 of the angular process. Left, molar zone; right, angular process.

The second step was to consider how, within each group (WLA, PWK, F1 and F2) the variation in one module was related to variation in other modules. The relationships within the parental groups were overall weak, possibly due to the reduced and unbalanced number of specimens (24 for PWK and 33 for WLA vs. 38 for F1 and 82 for F2 hybrids) and the sensitivity of inter-distance comparison to sampling [55]. The inter-individual pattern of variation for the alveolar region and the ramus were overall related, although more strongly in hybrids than in parental groups. Considering the partitioning of the mandible into five modules, significant co-variations emerged especially between neighboring modules (Figure 5b). Among these covariations, the relationship between the molar region and the angular process repeatedly emerged. These two regions are strongly related by the functional constraints of mastication. This relationship was exemplified in F1 hybrids (Figure 6), showing that an uplift of the upper molar zone is associated with a downward development of the angular process (Figure 6).

## Discussion

### A mosaic of modules in hybrids

Our results demonstrated how hybridization can quite simply produce new morphological variation in the case of a complex feature like the mandible that is composed of several parts with relative genetic and developmental independence. Each one of these parts is most probably under the control of a complex genetic network [25]. Accordingly, hybrids evidenced quite a disparate response to hybridization. In a first approximation, three patterns of response to hybridization were displayed, depending on the module considered. (1) Hybrids were characterized by an intermediate morphotype as expected under the simple additive genetic model. (2) Hybrids displayed a transgressive morphology as expected due multigenic interactions. Not surprisingly, this pattern was especially marked in F2 hybrids due to the effect of inter- and intra-chromosomal recombination. (3) Hybrids were close to one parental group, according to a pattern of ‘dominance’. Such patterns were surprising, because they would suggest that the shape of these modules should be mostly controlled by a single major gene or complex of gene, an unexpected results for traits known to be highly multigenic [22,25,26].

The overall morphology emerging from this mosaic was fully new, since it mixed parts derived from each of the parental strains, parts with an intermediate shape, and parts themselves transgressive. Accordingly, structures composed of several modules tended to show a stronger transgressive pattern when compared to single modules.

Such a mechanism requires no recombination between the two divergent genomes to produce transgressive phenotypes. Accordingly, the transgression was observed as soon as the first generation of hybrids [37]. Increased transgression and variance are nevertheless expected in the second-generation hybrids, because of (1) the effect of genetic recombination mixing the parental genomes, which apparently does exist for each module separately and (2) the number of possible combinations between the different modules which increases since recessive morphologies can also be expressed. Our results validate both predictions: transgression occurring as soon as F1 hybrids, but in a stronger and more variable way in F2 hybrids.

### A modular response to heterosis

Remarkably, such a modular response to hybridization not only characterizes the shape of each module, but also its size. Hybrids between the two subspecies of mice have been shown to display a hybrid vigor that translates not only into an increased developmental stability [34] but also into a larger overall size [43]. Accordingly, mandible size is larger in hybrids compared to parental strains [37] but surprisingly, this pattern does not hold true for each module. Hybrids tend to be of a similar size to the largest of the parents when one strain is obviously larger than the other for a given module (for instance, hybrids are similar in size to the *domesticus* parental strain in the case of the coronoid process, which is much larger than in the *musculus* parental strain). The heterotic effect of size increase for the hybrids only holds true for the F1 hybrids and F2 are mostly characterized, as for shape, by an increased variance covering the whole range of possible sizes, from the smallest to the largest of the parental strains, or to the largest F1 hybrids when these latter display a transgressive size. Such a modular size response to hybridization can contribute to produce new mandible morphologies without involving shape changes of each one of the modules.

The modularity in shape does not seem to be only an allometric by-product of the modularity in size. Modules showing closeness in size to a given strain are not systematically the same ones showing closeness in shape towards the same strain. Hybrids are indeed close to the *domesticus* parents for size and shape regarding the molar zone, but for the coronoid process, F1 hybrids are close to the *domesticus* size but have an intermediate shape between both parental strains. These results rather suggest that the genetic control of size and shape differ for each module, and that both modularity in size and shape can contribute to the increased morphological variation in hybrids, including the production of phenotypes out of the parental range.

### Remodeling as an adjustment between neighboring modules?

The mandible is not just a mosaic of morphological parts determined by genetic networks; it is also a feature of selective importance for the animal since it allows handling food. An efficient function is thus a crucial requirement for the evolutionary outcome of any morphological change. Even in controlled laboratory conditions where selective pressures are expected to be reduced since animals are fed *ad libidum*, a functional mandible is an evident requirement. The mosaic of disparate parts should thus be adjusted into a functional ensemble and this should lead to an integrated variation of the modules supposed to work together.

No general pattern of integration emerged from our data-set. Integration between modules tended to be low in the parental strains, and to relate modules from the same (anterior vs. posterior) part of the mandible, and/or neighboring modules. Beyond these discrepancies, a significant covariation of the anterior and posterior parts of the mandible, especially due to the integration of the angular process with the molar zone, repeatedly emerged in all parental groups as well as in hybrids. The angular process is the zone of insertion of the masseteric muscles that are the most important in moving the mandible for the occlusion of molar teeth [56]. Their integration is thus a functional expectation, and indeed has been shown to emerge repeatedly, from within-species to among-genera evolutionary level [31]. The occurrence of this covariation in the particular case of hybrids suggests of a process ensuring the adjustment of parts made independent by the modular response to hybridization.

The adjustment of the modules into a functional mandible is probably insured by bone remodeling. The importance of this process in shaping structures such as the mandible has been widely underestimated, since quantitative studies were usually based on adult animals supposed to have more or less reached a definitive morphology. Yet, mice only reach about 80% of their adult skull size at weaning [57], a value that still allows a significant growth. Bone remodeling modifying the mandible shape may occur during this late growth, as demonstrated by significant mandible shape differences as response to differences in food consistency [58,59] or functioning in the context of a muscular dystrophy [60,61]. Accordingly, it has been suggested that parts of the mandible functioning with the same muscles form an integrated complex and that bone remodeling tends to modify the mandible as a whole, beyond its developmental and genetic modularity [62]. Our results support such an adjustment role allowing the integration of such a genetic modular mosaic into a functional mandible.

The example of the mandible as a whole shows that choosing a trait that is indeed composed of several modules, such as the mandible, as a character of interest may blur the interpretation of the processes underlying the transgressive effects. Surprisingly for morphological traits known to be under multigenic determinism, several modules responded to hybridization in a way analogue, in a first approximation, to a simple monogenic determinism, showing additive or ‘dominance’ pattern. Considering them together obscures such intriguing patterns and transgression emerges as the characteristic signature of hybrids, in agreement with the expectations of a complex multigenic determinism [26]. Conversely, the functional integration that takes place between modules may lead to an increase of transgressive signatures due to the fact that modules are submitted to non-genetic influences from other surrounding modules and structures (such as muscles), a process that may hide the basic genetic signal of each separate module.

## Conclusions

Long-term evolutionary outcomes of modularity have been set forth, allowing the reorganization of the different components of the mandible depending on the changes in the functional demands during the course of evolution [31] as well as the evolution of the phenotype-genotype relationship and modularity itself [63]. Our results illustrate another way of how modularity can generate new morphological variation. The relative genetic independence of the different modules simply produces new combinations when two genomes are brought into contact by a hybridization process. Furthermore, this process may cause intriguing patterns of divergence even with a little percentage of introgression. Since the importance of hybridization on the wild is increasingly recognized [64,65], this process may be a significant factor maintaining and generating morphological diversity even among groups separated by overall little genetic divergence.

## Competing interests

The author declare that they have no competing interests.

## Authors’ contributions

SR, PA and JCA designed the study and wrote the manuscript. PA was in charge of the breeding of the animals. SR measured the samples and performed the statistics. All authors read and approved the final manuscript.
